# Increase in immune cell infiltration with progression of oral epithelium from hyperkeratosis to dysplasia and carcinoma

**DOI:** 10.1038/sj.bjc.6600282

**Published:** 2002-05-06

**Authors:** G Gannot, I Gannot, H Vered, A Buchner, Y Keisari

**Affiliations:** Department of Oral Pathology and Oral Medicine, The Maurice and Gabriela Goldschleger School of Dental Medicine, Tel-Aviv University, Tel Aviv, Israel; Department of Human Microbiology, Sackler Faculty of Medicine, Tel-Aviv University, Tel Aviv, Israel; Department of Bio-medical Engineering, Faculty of Engineering, Tel-Aviv University, Tel Aviv, Israel

**Keywords:** transformation, carcinoma, immune infiltrate, human, diagnosis

## Abstract

In the present study, epithelium derived lesions of various pathological manifestations were examined histologically and immunohistochemically for mononuclear cell infiltration. The infiltrate under the transformed epithelium of oral lesions, was examined for differences in the composition of immune mononuclear cells as the epithelium moves from hyperkeratosis through various degrees of dysplasia to squamous cell carcinoma. The study was performed on 53 human tongue tissues diagnosed as hyperkeratosis (11 cases), mild dysplasia (nine cases), moderate and severe dysplasia (14 cases) and squamous cell carcinoma (19 cases). A similar analysis was performed on 30 parotid gland tissues diagnosed as pleomorphic adenoma (14 cases) and carcinoma ex-pleomorphic adenoma (16 cases). Immunohistochemical analysis of various surface markers of the tumour infiltrating immune cells was performed and correlated with the transformation level as defined by morphology and the expression of p53 in the epithelium. The results revealed that, in the tongue lesions, the changes in the epithelium from normal appearance to transformed were accompanied by a corresponding increase in the infiltration of CD4, CD8, CD14, CD19+20, and HLA/DR positive cells. The most significant change was an increase in B lymphocytes in tongue lesions, that was in accordance with the transformation level (*P*<0.001). In the salivary gland, a significant number of cases did not show an infiltrate. In cases where an infiltrate was present, a similar pattern was observed and the more malignant tissues exhibited a higher degree of immune cell infiltration.

*British Journal of Cancer* (2002) **86**, 1444–1448. DOI: 10.1038/sj/bjc/6600282 www.bjcancer.com

© 2002 Cancer Research UK

## 

Oral squamous cell carcinoma (SCC) causes 2% of all cancer-related deaths (Shugars and Patton, 1997). The long-term prognosis of newly diagnosed SCC remains poor, with an overall 5-year survival rate of only 50% (Prince and Bailey, 1999). Much of the morbidity and mortality associated with oral cancer can be prevented or limited by early detection. However, early lesions are difficult to diagnose because they are usually asymptomatic, vary greatly in clinical appearance and are easily misdiagnosed (Partridge *et al*, 1997).

Transformation occurs through various changes in the epithelium. The initial step towards a premalignant lesion is a reversible step of hyperkeratosis, where morphologically the epithelium thickens and an excess of keratin is observed on its outer layer. Further morphological changes are the different stages of dysplasia. Mild dysplasia is the epithelial transformation in the lower layers of the epithelium, whereas moderate and severe dysplasias are the transformation in half to full thickness of the epithelium. Histologically, carcinoma is an invasive epithelium in the connective tissue (Liu and Klein-Szanto, 2000).

In the present study, an attempt was made to establish the immune cell infiltrate profile at different stages of oral lesions and to correlate it with the progression from normal appearing epithelium through dysplasia into carcinoma. The tongue was the examined area, since it is the most common site of oral carcinoma (Prince and Bailey, 1999). To further assess the phenomenon of immune cell infiltration in tumours of epithelial origin, we also examined the infiltrate profile in Pleomorphic Adenomas (PA) in the parotid gland. PA, the most common benign salivary gland neoplasm (Ellis and Auclair, 1996), is an epithelial origin tumour that accounts for 53–77% of the parotid tumors (Neville *et al*, 1995). With adequate surgery, the prognosis is excellent, with a cure rate of more than 95% (Neville *et al*, 1995). There is evidence that the carcinoma ex-pleomorphic adenoma (C/PA) represents a malignant transformation within PA (Yamamoto *et al*, 1996; Li *et al*, 1997), with a death rate of 50% for invasive lesions. C/PA lesions were examined for immune cell infiltration and compared to the benign counterpart.

Immunohistochemistry was carried out on 53 tongue lesions diagnosed as hyperkeratosis, mild, moderate and severe dysplasia, and SCC, and on 30 parotid gland tissues diagnosed as PA or C/PA.

All tissue samples were analysed for the presence of cells with surface markers of T cells, B cells and monocytes in the immune infiltrate. The extent and cellular profile of the infiltrate was correlated with the transformation level that was established by morphology and p53 expression of the epithelium.

## MATERIALS AND METHODS

### Selection of cases

For the study, 53 formalin fixed and paraffin embedded blocks of the lateral surface of the tongue and 30 blocks of the parotid gland were obtained from the Department of Oral Pathology (Tel Aviv University and Rabin Medical Center, Petach-Tikvah, Israel), and from the Armed Forces Institute of Pathology (AFIP) (Washington DC, USA). Certified oral pathologists graded the cases, which were grouped by morphology. In the tongue: hyperkeratosis (11 cases), mild dysplasia (nine cases), moderate and severe dysplasia (14 cases) and squamous cell carcinoma (19 cases); in the parotid gland: PA of the parotid gland (14 samples) and C/PA of the parotid gland (16 samples).

### Immunostaining

The blocks were cut to 3 μm thickness and reacted with various antibodies according to the immunohistochemistry protocol (Zymed Histostain-Plus Kit, South San-Francisco, CA, USA). Briefly, sections were pretreated with 3% methanol and hydrogen peroxide solution, and then washed twice with double distilled water. Special pre-treatments were carried out according to the manufacturer's instructions.

Sections were stained immunohistochemically using the Zymed broad spectrum kit (Zymed, CA, USA) and antibodies against the following molecules:

1. CD4 (ZYMED, CA, USA. Cat No: 08-0101) for detection of T helper cells
2. CD8 (DAKO, CA, USA. Cat No: N1592) for detection of T cytotoxic cells
3. CD14 (ZYMED, CA, USA. Cat No: 08-0121) for detection of monocytes/macrophages
4. HLA/DR (DAKO, CA, USA. Cat No: 060-061) for detection of antigen presenting cells.
5. CD19+20 (PanB. ZYMED, CA, USA. Cat No: 08-0088) for detection of B cells.
6. p53 (DAKO, CA, USA. Cat No: M7001) for detection of wild type and mutated p53 antigen in epithelial cells.

The stained sections were photographed (a constant area of 0.04 mm^2^) using a magnification of ×200 in an Olympus microscope. Four pictures were taken from each slide at different areas. The pictures were scanned and analysed for total number of cells and for positive (stained cells) and negative (not stained) cells using a computer program (ImagePro 4.0, Cybernetics, Silver Spring, MD, USA), and a mean was calculated for the values of each specimen. Results were expressed as the mean of positive cells/Total (positive+negative cells) in the infiltrate for lymphocyte markers, CD14 and HLA/DR or in the epithelium for p53.

Statistical analysis was performed using one-way ANOVA test.

## RESULTS

### Immune cell infiltration in lesions of the tongue

Evaluation of the inflammatory infiltrate under lesions of hyperkeratosis, dysplasia and SCC in the human tongue revealed that the amount of the infiltrating cells was significantly higher in moderate and severe dysplasia and SCC compared to hyperkeratosis and mild dysplasia. In a 0.04 mm^2^ area, only 102±15 and 82±24 infiltrating cells were counted in hyperkeratosis and mild dysplasia, respectively. The difference between the two groups was non-significant. The number of infiltrating cells was significantly elevated in cases of moderate and severe dysplasia and SCC (170±27 and 176±30 cell, respectively) compared with the hyperkeratosis and mild dysplasia groups (*P*<0.05, Table 1

**Table 1:**
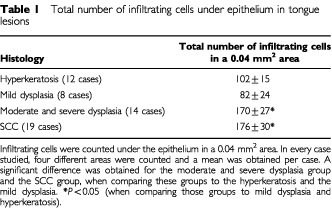
Total number of infiltrating cells under epithelium in tonguelesions

). There was no significant difference between the group of moderate and severe dysplasia and SCC.

**Figure 1:**
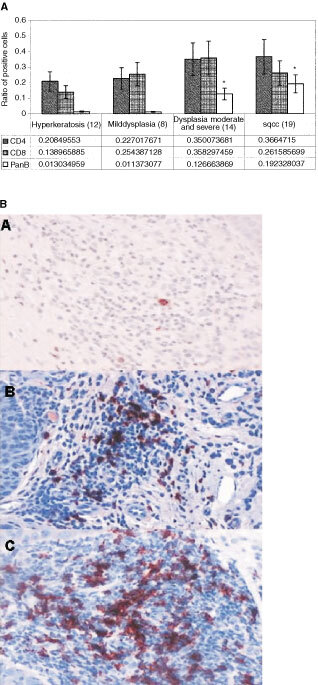
(**A**) Lymphocyte profile of tongue lesions. Immunohistochemical detection of lymphocyte markers. Sections were reacted with antibodies for CD4, CD8 and CD19+20 using an immunohistochemical protocol. Positive cells were counted out of a total number of infiltrating cells in a 0.04 mm^2^ area. The bars represent the percentage of positive cells counted out of total number of infiltration cells. In every case four different areas were counted and a mean was established per case. In parentheses are the number of cases for each group. *Significant difference between these groups when compared to hyperkeratosis and mild dysplasia. (**B**) Immunohistochemical staining of tongue lesions with B cell antibody. The red staining represents positive reaction. (**a**) Hyperkeratosis, (**b**) moderate and severe dysplasia, (**c**) SCC. Demonstration of B cells (stained in red) in cases of hyperkeratosis, moderate and severe dysplasia and SCC. Notice the dramatic elevation in the number of B cells when the epithelium is dysplastic (in the cases of moderate and severe dysplasia and SCC). Magnification of ×200.

Immunohistochemical analysis of the tongue infiltrating cells, revealed a general trend of increase in the proportion of T cells, B cells, and macrophages as the epithelial lesions changed towards a more malignant phenotype. The total population of CD4, CD8 and B cells, in the tissue specimens, were evidently higher in moderate and severe dysplasia and SCC lesions compared with hyperkeratosis and mild dysplastic lesions. A significant difference was found in the proportion of B-cells in the hyperkeratosis and mild dysplastic lesions compared with moderate and severe dysplasia and SCC lesions (*P*=0.004, Figure 1A

). B-cells, in the tongue infiltrate, were very prominent in moderate and severe dysplasia and even more so in the SCC specimens (Figure 1B).

**Figure 2:**
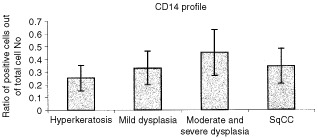
CD14 cell profile of tongue lesions. Immunohistochemical detection of CD14 positive cells. Sections were reacted with an antibody for CD14 using an immunohistochemical protocol. Positive cells were counted out of a total number of infiltrating cells in a 0.04 mm^2^ area. The bars represent the percentage of positive cells counted out of total number of infiltrating cells. In each case four different areas were counted and a mean was established per case.

The interaction of the infiltrating cells with antibodies to CD14 and HLA/DR also indicated that a higher proportion of positive cells correlated with advanced epithelial transformation. There was an increase in the CD14 positive cells in the infiltrate with malignant transformation of the epithelium, however, the amount of CD14 positive cells decreased slightly when the epithelium transformed to carcinoma (Figure 2

). The differences in CD14 positive cells were not statistically significant (*P*=0.181).

**Figure 3:**
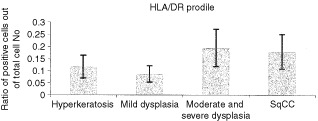
HLA/DR profile of tongue lesions. Immunohistochemical detection of HLA/DR positive cells. Sections were reacted with an antibody for CD14 using an immunohistochemical protocol. Positive cells were counted out of a total number of infiltrating cells in a 0.04 mm^2^ area. The bars represent the percentage of positive cells counted out of total number of infiltrating cells. In each case four different areas were counted and a mean was established per case.

A similar pattern was observed for HLA/DR positive cells when comparing lesions of SCC and moderate and severe dysplasia to lesions of hyperkeratosis and mild dysplasia (Figure 3

). The differences, though evident, were not significant (*P*= 0.39).

**Figure 4:**
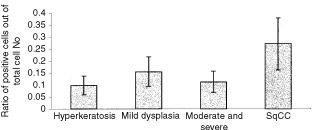
p53 profile of epithelium in tongue lesions. Immunohistochemical detection of p53 positive epithelial cells. Sections were reacted with an antibody for p53 using an immunohistochemical protocol. Positive epithelial cells were counted in a 0.04 mm^2^ area out of a total number of epithelial cells. The bars represent the percentage of positive cells counted out of total number of epithelial cells. In each case four different areas were counted and a mean was established per case.

**Figure 5:**
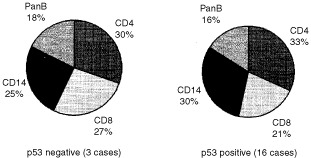
Profile of infiltrating cells according to p53 staining in SCC tissues of the tongue. Cases of SCC were grouped according to their staining for p53. The cell infiltrate profile of three cases of negative p53 epithelial staining was compared to these of the epithelial p53 positive SCC cases.

All tongue lesions were also stained immunohistochemically with anti p53 antibodies to detect accumulation of wild type or mutated p53 in epithelial cells. An increase was found in the p53 positive cells in SCC lesions compared to dysplasia and hyperkeratosis (Figure 4

). When cases were divided into p53 positive and p53 negative, no significant difference was found in the composition of the cellular infiltrate between the samples in all four groups examined (data shown for SCC group only, Figure 5

).

### Immune cell infiltration in lesions of the parotid gland

Immunohistochemistry was applied to a different site in the head and neck region, for evaluation of the inflammatory/immune infiltrate. PA and C/PA lesions of the parotid gland were examined for the immunological profile of infiltrating cells. These lesions were heterogeneous with respect to the presence of a mononuclear cell infiltrate. No cell infiltrate was observed in 38% of PA and 35% of C/PA lesions. In positive lesions, a mean of 152±40 cells was found in PA, while 242±100 cells were counted in sections of C/PA. This increase was statistically significant (*P*<0.05).

**Figure 6:**
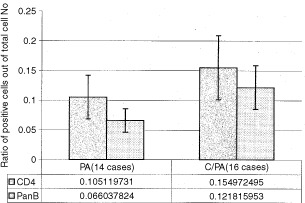
A summary of parotid gland results. Sections were reacted with antibodies for CD4 and CD19+20 (B cells) using an immunohistochemical protocol. CD 4 reacted positive with nine out of 14 cases of PA and 12 out of 16 cases of C/PA. Positive staining for CD19+20 was found in eight out of 14 cases of PA and eight out of 16 cases of C/PA. In each case four different areas (0.04 mm^2^) were counted and a mean was calculated per case. The bars represent the percentage of positive cells counted out of a total number of infiltrating cells in cases positive for the antibody examined.

When the tissues were stained immunohistochemically with antibodies to immune cell markers, no cells positive for CD8 or CD14, were observed. Reacting the tissues with anti CD4 and B cell antibodies revealed an increase in the ratio of positive cells when the lesion transformed to malignancy (Figure 6

). There was no difference in the ratio of positive cells for HLA/DR between PA and C/PA lesions (0.145±0.04 positive cells out of total infiltrating cells for PA and 0.147±0.05 positive cells out of total infiltrating cells for C/PA).

CD4 positive cells were observed in nine out of 14 cases of PA and 12 out of 16 cases of C/PA. B cells were found in eight out of 14 cases of PA and eight out of 16 cases of C/PA. Anti HLA/DR antibodies gave positive results only in three out of 14 cases of PA and five out of 16 cases of C/PA. Reacting the epithelial component of the parotid lesions with anti p53 antibodies revealed an increase in the ratio of positive epithelial cells with transformation (0.015±0.005 positive cells in PA and 0.12±0.04 positive cells in a 0.04 mm^2^ area in C/PA).

## DISCUSSION

SCC is an immunogenic tumour, arising against a background of cellular immunodeficiency with lymphopenia, decreased T cell and macrophage function and anergy, and it has been suggested that there is deterioration in the immunologic status in SCC (Richtsmeier, 1997). Nevertheless, immunotherapeutic agents have been used to treat SCC. In a phase two trial of SCC of head and neck patients treated with a natural cytokine mixture, that was expected to stimulate anti tumour specific immune responses, lesions were reduced in size and in some patients completely disappeared (Barrera *et al*, 2000).

Inflammatory mononuclear cell infiltrates were found in association with oral premalignant lesions and SCC (Migliorati *et al*, 1986). CD4 positive cells increased in number when the lesion became malignant as compared to normal mucosa and premalignant lesions (Hirota *et al*, 1990; Vetto *et al*, 1997).

In the present study we further inquired the interrelationship between oral lesion, including SCC, and the immune response. The cellular composition of cells infiltrating oral epithelium lesions was systematically evaluated, and correlated with the level of transformation of the epithelium. The mononuclear cells in the infiltrate were examined and thoroughly counted to establish an accurate profile for the immune infiltrate in various stages of epithelial transformation.

The general finding was that severe pathological changes in epithelial tissues (i.e. moderate and severe dysplasia or SCC) are accompanied by a higher level of infiltrating lymphocytes and macrophages, when compared to lesions with milder changes (i.e. hyperkeratosis or mild dysplasia).

Steps towards malignancy had a distinct lymphocyte profile. As the lesion became malignant, the total amount of infiltrating cells increased and the population of the infiltrating cells changed from non-specialized infiltrating cells to T and B lymphocytes. This change was gradual as the lesion became more malignant, and it peaked in the moderate and severe dysplastic lesions. B lymphocytes became significantly prominent in severe dysplasia and SCC. An interesting finding was the different profile of the immune cells between mild dysplastic lesions and moderate and severe dysplasia. The infiltrate in mild dysplasia was similar to hyperkeratosis in the population of the B lymphocytes, although T cells and CD14 positive cells were more abundant in mild dysplasia.

Rita *et al* (1996) examined head and neck cancers for immune inhibitory factors and found that IL-10, together with TGF-β and prostaglandines, were associated with a reduced content of CD8+ cells. In the present study, the amount of CD8+ cells in SCC was lower than CD8+ cells in the moderate and severe dysplastic lesions, which might indicate that the malignant lesion is less attractive for CD8+ cells or that the cytotoxic response was reduced in the malignant state.

The lymphocyte profile was also examined in epithelial cell derived lesions in the parotid gland, where the environment is completely different from the tongue (Ito *et al*, 1999). In parotid gland lesions, as in tongue lesions, the shift of the lesions towards malignancy and its correlation with mononuclear cell infiltration, were examined. Since PA are encapsulated lesions, they do not attract a pronounced infiltrate (Ikeda *et al*, 1996; Zhao *et al*, 1999). Even when the lesion changes to carcinoma, it is still partly encapsulated (Auclair and Ellis, 1996). It is difficult to draw final conclusions regarding parotid lesions because in a substantial number of the cases no infiltrate was observed. As in the tongue lesions, the change from the benign (PA) to the malignant form (C/PA) was accompanied by an increase in CD4 and B cells, but not in HLA/DR positive cells. It is important to note that CD8 and CD14 positive cells were not observed in parotid gland lesions.

Recent studies focused on p53 in carcinomas (Mitsunaga *et al*, 1995; Lavieille *et al*, 1996; Ralhan *et al*, 2000). Antibodies have been found in the blood of patients for p53 proteins and its mutated variants. In the present study tongue lesions with the SCC phenotype exhibited a higher expression of p53 when compared to dysplastic or hyperkeratotic lesions. However, when p53 positive and p53 negative cases were compared, they both had similar levels of immune infiltrating cells.

The present data showed that there was an increased tendency of mature immune cells, especially of B cells, to infiltrate the more malignant epithelial lesions.

These findings may be important for diagnostic purposes when there is a problem defining the nature of the epithelial transformation. However, more cases are needed to establish an accurate pattern of the cell infiltrate in malignant lesions, and the most significant cell type(s).
